# A Case of Fibroelastoma with Widespread Embolism to the Brain, Kidney, and Spleen

**DOI:** 10.7759/cureus.4798

**Published:** 2019-06-01

**Authors:** Iqra Iqbal, Waqas Ullah, Muhammad Atique Alam Khan, Shujaul Haq, Muhammad Arslan Cheema

**Affiliations:** 1 Internal Medicine, Abington Hospital-Jefferson Health, Abington, USA; 2 Internal Medicine, Mayo Hospital, King Edward Medical University, Lahore, PAK

**Keywords:** renal embolism, splenic embolism, fibroelastoma, stroke

## Abstract

Cardiac papillary fibroelastoma (CPF) is the second most common primary cardiac tumor, which is diagnosed incidentally or with embolic phenomena, mostly in the form of a transient ischemic attack (TIA) and stroke. We present a case of a 58-year-old female who presented with fatigue and low-grade fever and was found to have multiple systemic infarcts. Her blood cultures and transthoracic echocardiography (TTE) were negative, ruling out infective endocarditis. However, transesophageal echocardiography (TEE) revealed a mobile mass at the aortic valve. The mass was surgically removed, and the aortic valve was repaired. The histological examination of the mass finally revealed a papillary fibroelastoma. To our knowledge, this is the first reported case where fibroelastoma presented with splenic and renal infarcts in combination with the cerebral infarcts. Since cardiac fibroelastoma can cause embolization to the cerebral, splenic, and renal vessels, we, therefore, advocate that it should be considered as one of the possible causes of widespread embolism. We also stress upon the importance of doing TEE in case of a suspected cardiac mass, as the TTE is more likely to give false-negative results.

## Introduction

Cardiac fibroelastoma is notorious for causing transient ischemic attack (TIA) or stroke due to embolization of its fragments to cerebral arteries, but embolization to splenic and renal vasculature has rarely been reported. We are publishing a unique constellation of all these three systems together, in a 58-year-old female. We think that it is essential to keep this differential diagnosis in mind when encountering a case of multiple emboli. It also highlights the importance of doing transesophageal echocardiography (TEE) in case of suspected cardiac mass. Our patient did not have any recurrence of embolic symptoms after the surgical resection of fibroelastoma.

## Case presentation

A 58-year-old female presented with complaints of visual spots, right arm and right leg weakness, and some difficulty in speaking. Her past medical history included coronary artery disease status post percutaneous intervention, hyperlipidemia, and hypertension on optimal medications such as aspirin, lisinopril, metoprolol, and pravastatin There have been no prior neurological problems except for a similar episode of visual spots one month ago, which resolved on its own. Her family history was significant for coronary artery disease in her father. She quit smoking a month ago after being an active smoker for more than 20 years. Her neurological examination was significant only for right upper extremity weakness. Sensations were intact except for right-sided extinction. Babinski sign was negative, and reflexes were normal. The rest of the systemic examination was unremarkable. Her laboratory investigations revealed normal liver and kidney functions. The complete blood count, HBA1c, and lipid profile were also within the normal range. Her computed tomography (CT) head without contrast revealed an abnormal density in the parietal distribution of the left middle cerebral artery (MCA), consistent with an infarct. Magnetic resonance aortography (MRA) was consistent with significant stenosis of the right vertebral artery and bilateral internal carotid arteries, more on the left side (74%). TTE was negative for any vegetations. The patient had left carotid endarterectomy and was discharged home on two antiplatelet drugs and a high dose of statin.

**Figure 1:**
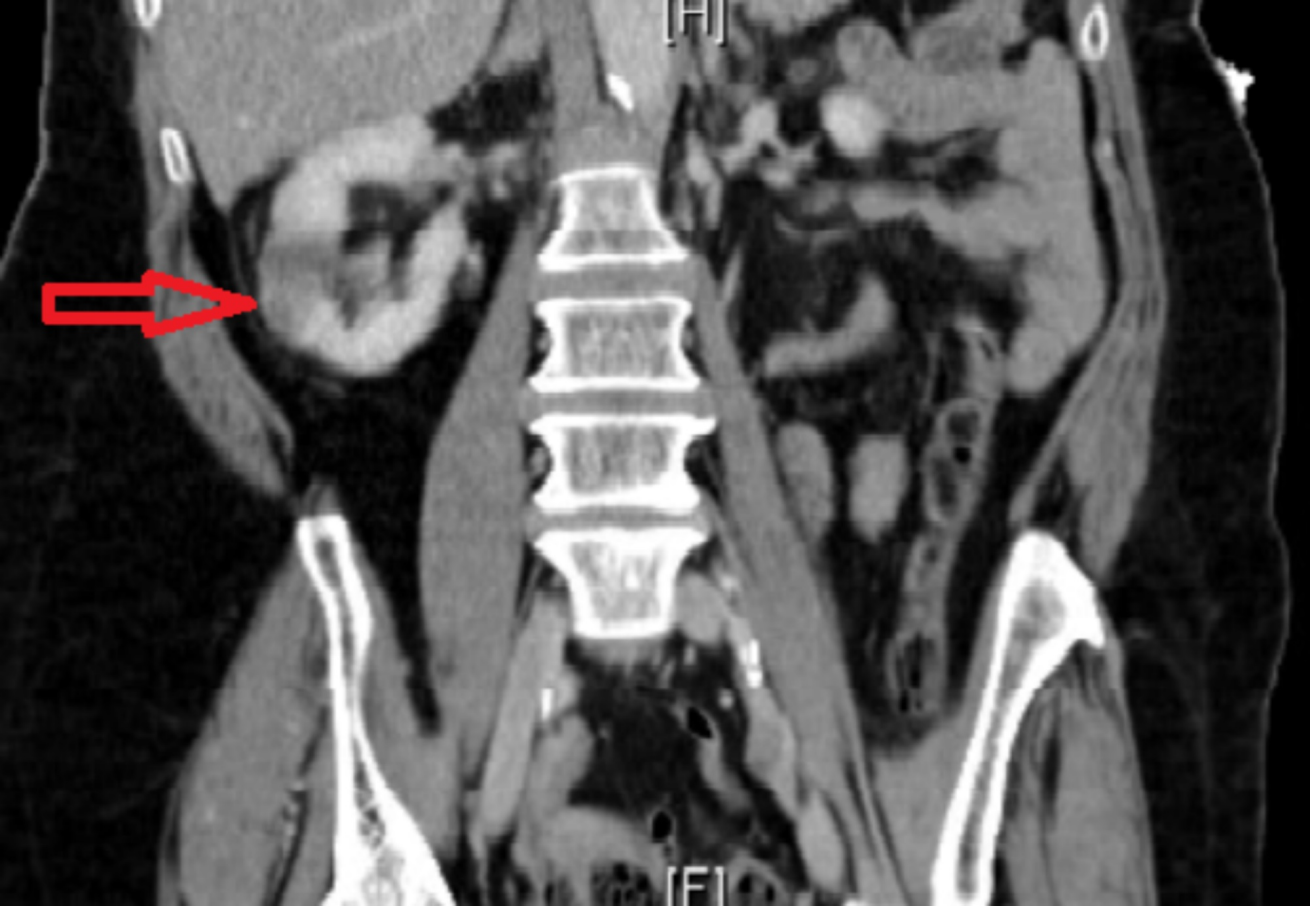
Computed tomography (CT) scan of the abdomen showing right renal infarct

**Figure 2:**
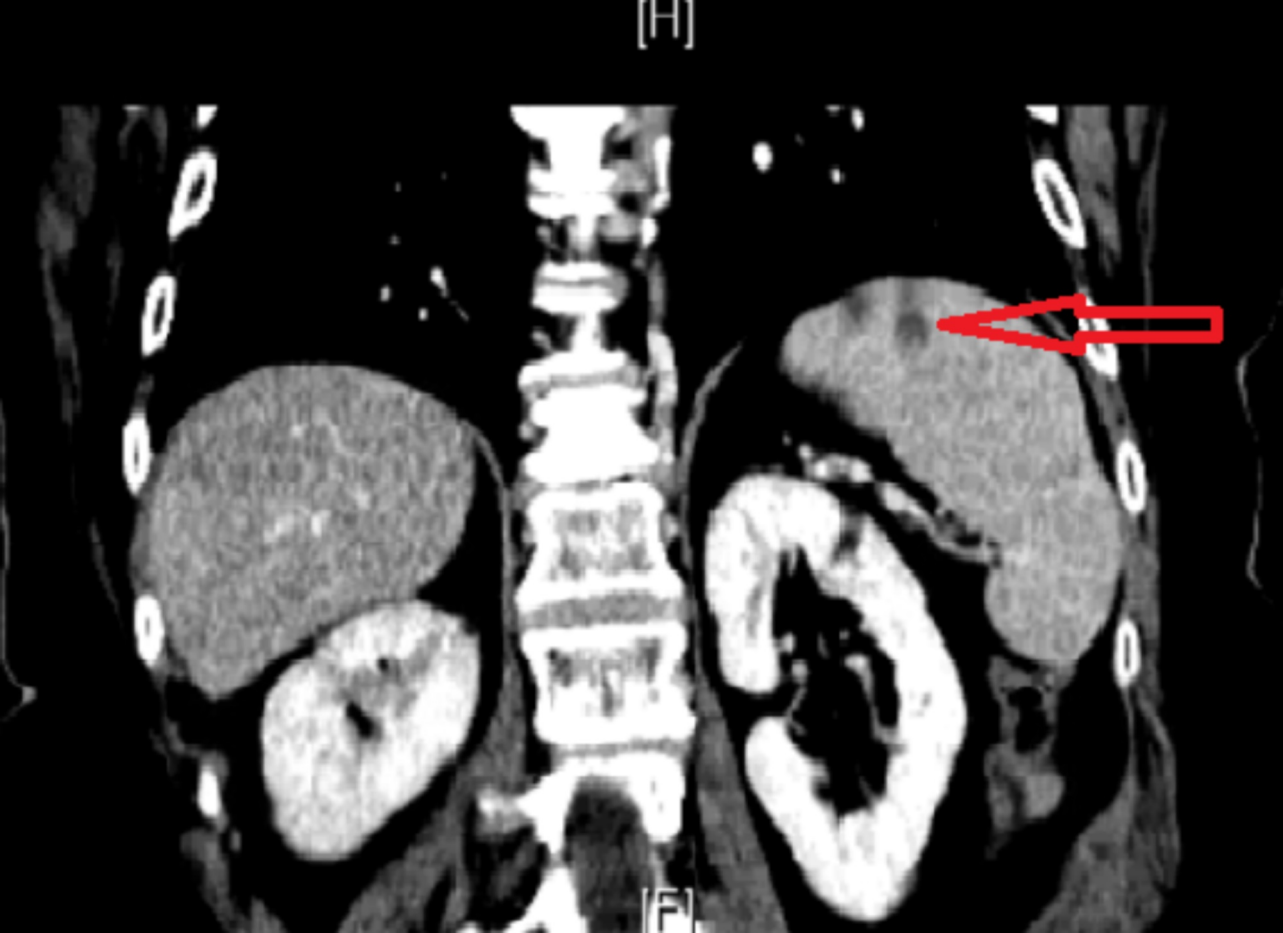
Computed tomography (CT) scan of the abdomen showing the splenic infarcts

**Figure 3:**
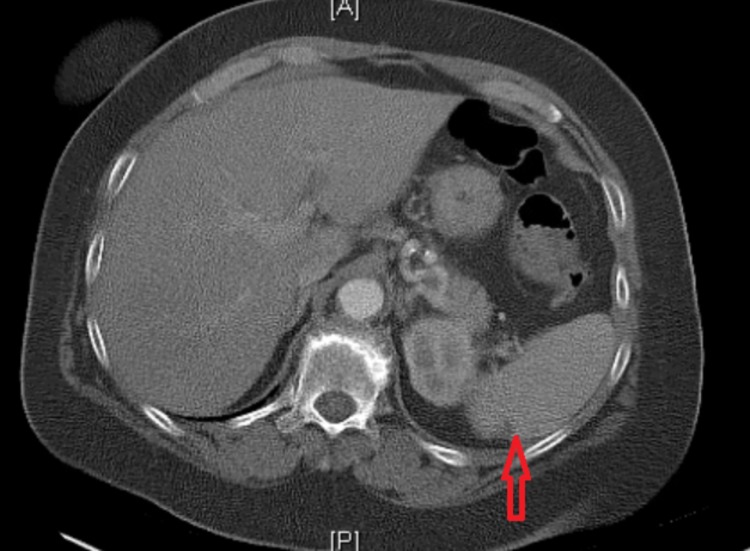
Computed tomography (CT) scan of the abdomen showing a splenic infarct

Two months later, she had a similar presentation of visual floaters and headaches associated with right flank pain, nausea, and low-grade fever. She denied any urinary complains, constipation, diarrhea, or a history of trauma. Neurological examination at this point revealed right arm pronator drift and right arm dysmetria. CT abdomen showed right renal indeterminate hypodense focus and few splenic hypodense foci (Figures 1-3).

**Figure 4:**
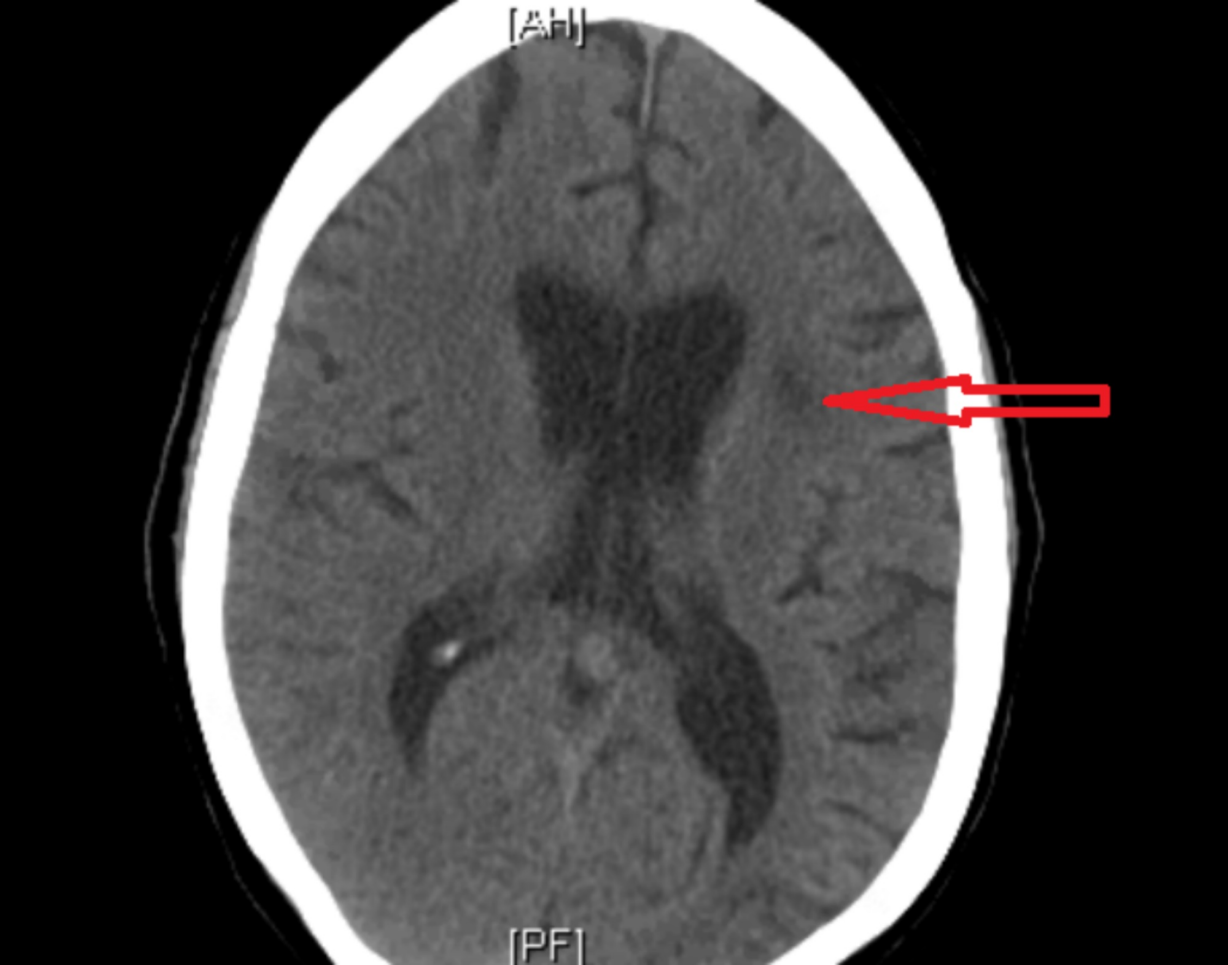
Computed tomography (CT) scan of the brain showing left temporal lobe infarct

The blood cultures and lumbar MRI done for the workup of fever were negative. CT brain revealed old left-sided infarcts. There was also an infarct of indeterminate age in the left internal capsule, which appeared different than previous imaging scans (Figure 4).

**Figure 5:**
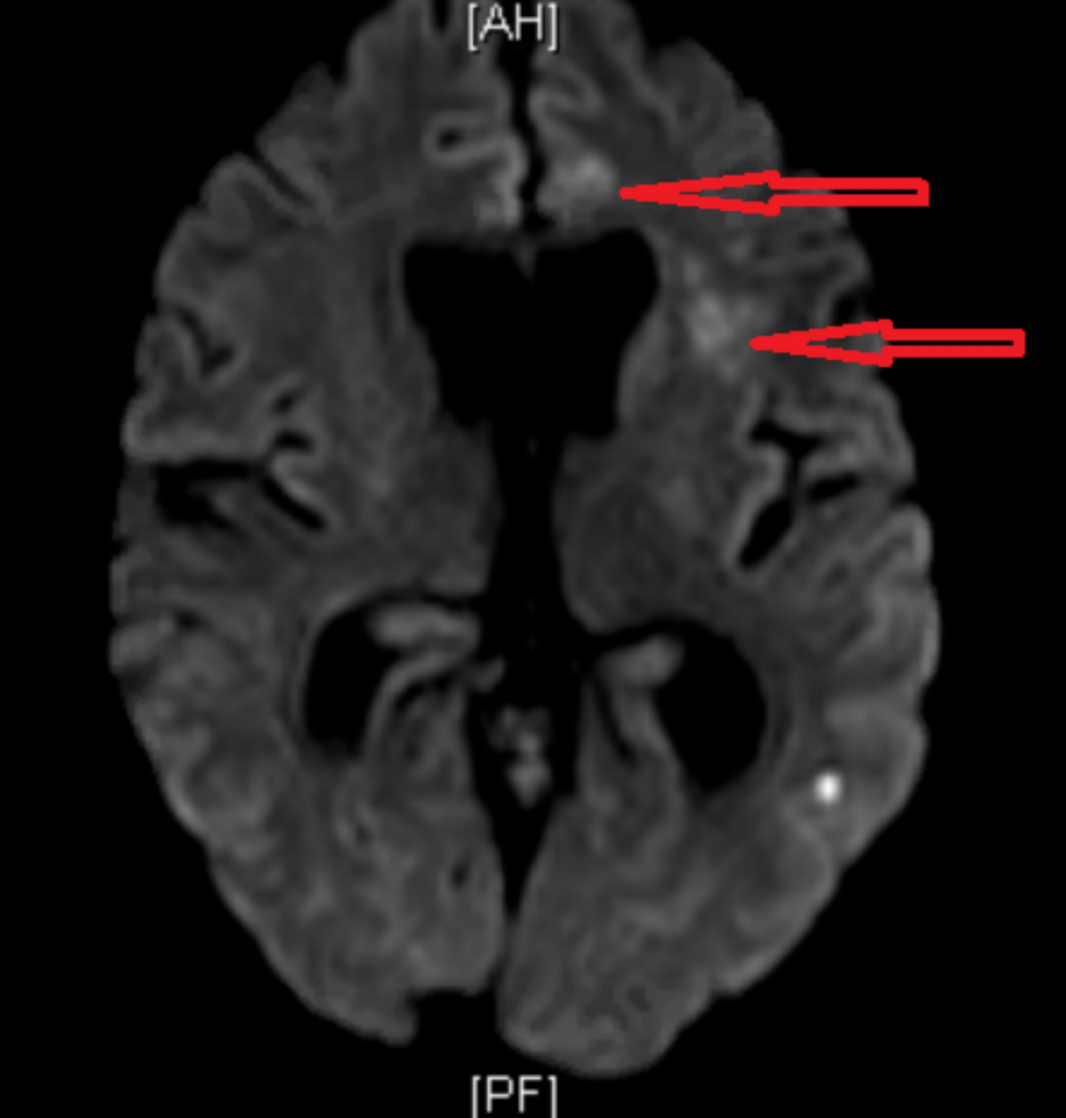
Magnetic resonance imaging (MRI) of the brain showing a left frontal and temporal lobe infarct

MRI brain showed punctate foci due to acute infarcts in the left cerebellum, left temporal lobe, right frontal, and left frontal lobe. These lesions were not present in the previous MRI (Figure 5).

**Figure 6:**
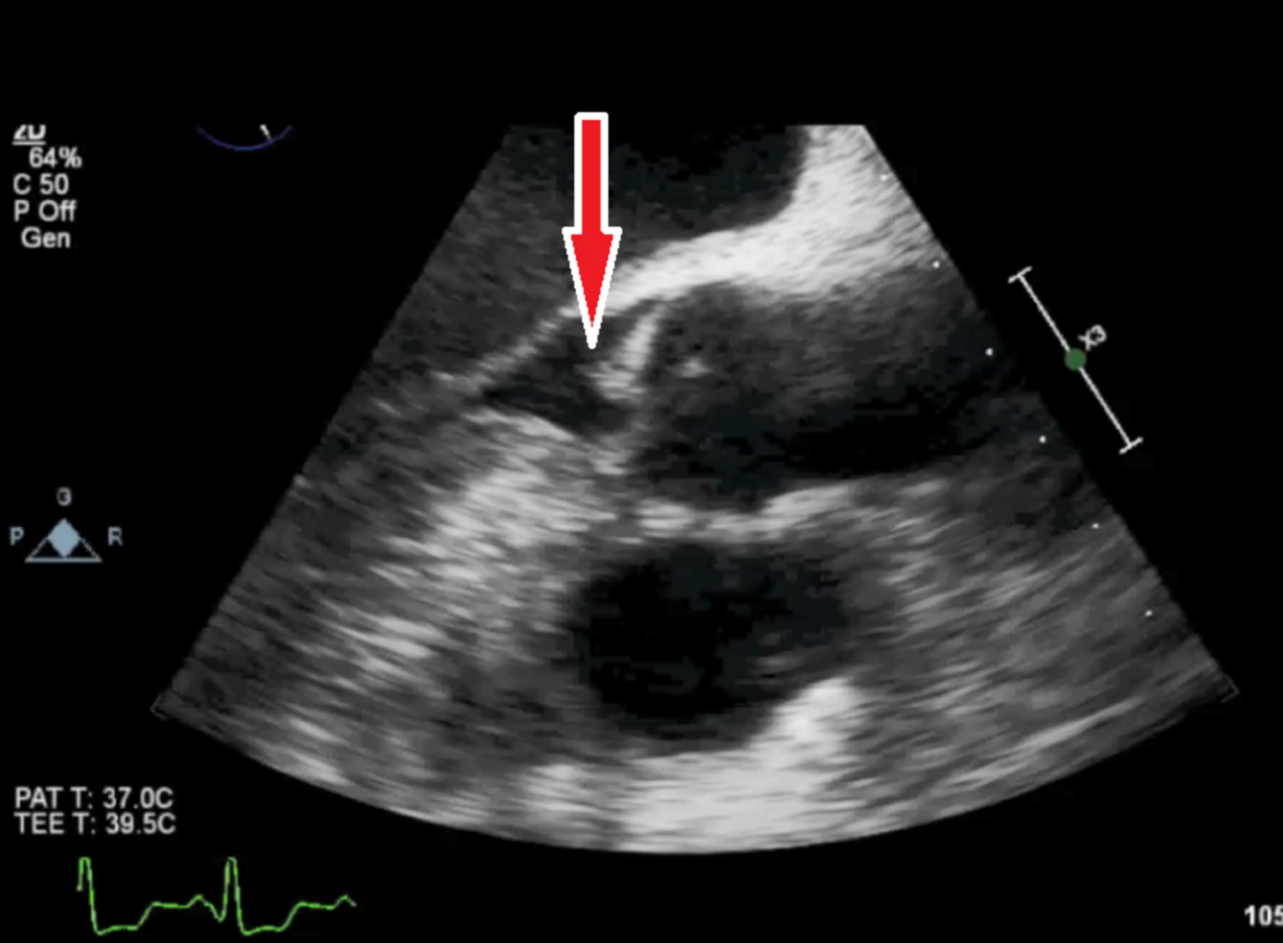
Transesophageal echocardiography (TEE) showing mass attached to the cusp of the aortic valve

TEE was performed, which revealed a small mobile mass on the right coronary cusp of the aortic valve with no vegetations (Figure 6).

**Figure 7:**
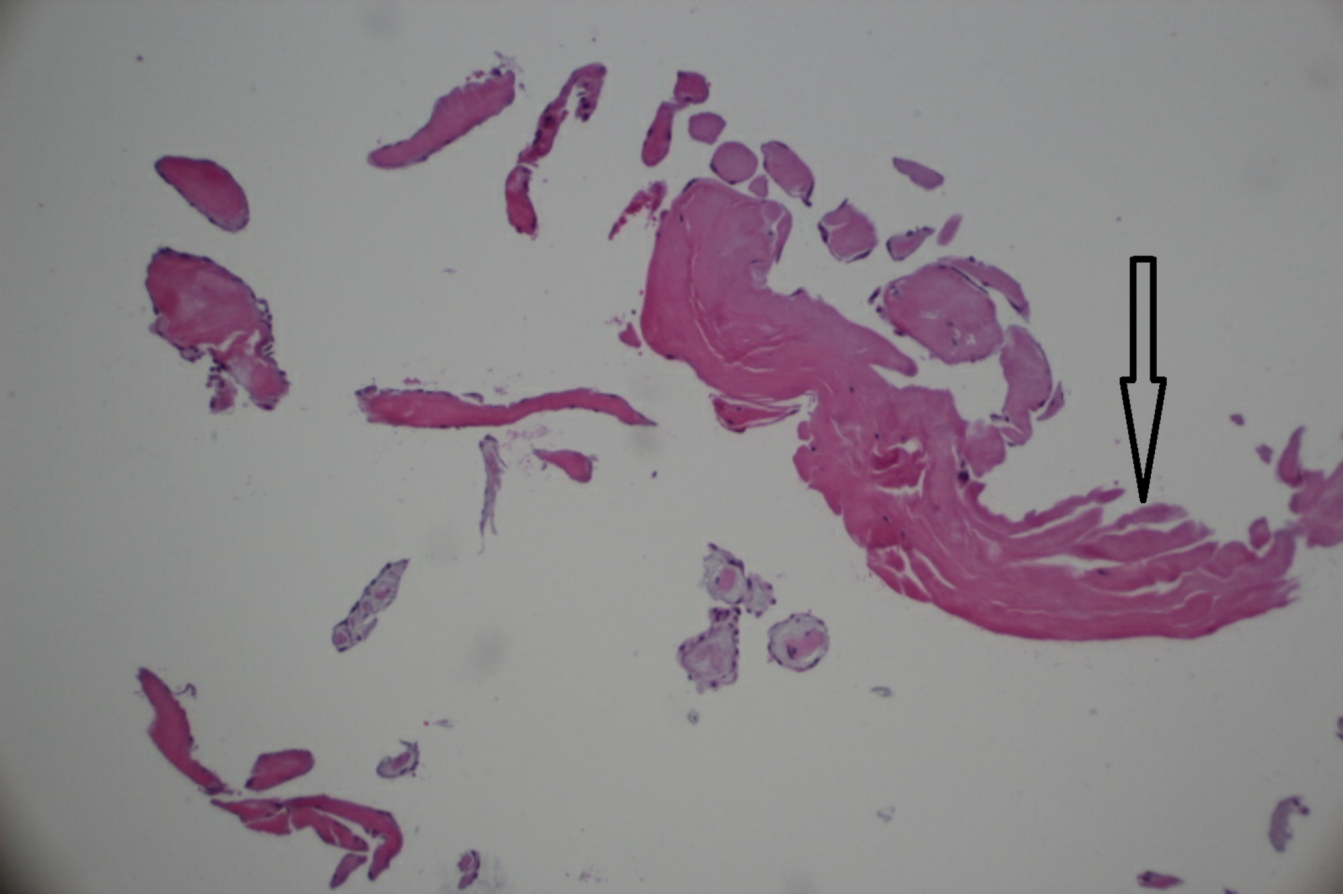
Microscopic view of the finger-like papillae in papillary fibroelastoma

The patient was initially started on empiric antibiotics, considering infection with culture-negative bacterial endocarditis. She then had a right anterior thoracotomy procedure done in the same admission, for excision of aortic valve mass with the repair of the aortic valve. There was a small mobile mass attached with a thin stalk to the right coronary cusp of the aortic valve. The resected specimen sent to the pathology lab was 0.6 x 0.2 x 0.1 cm in size. The result of the histopathological evaluation was reported as cardiac papillary fibroelastoma (CPF). It showed papillary projections under the microscope, with endothelium lining the surface (Figure 7).

**Figure 8:**
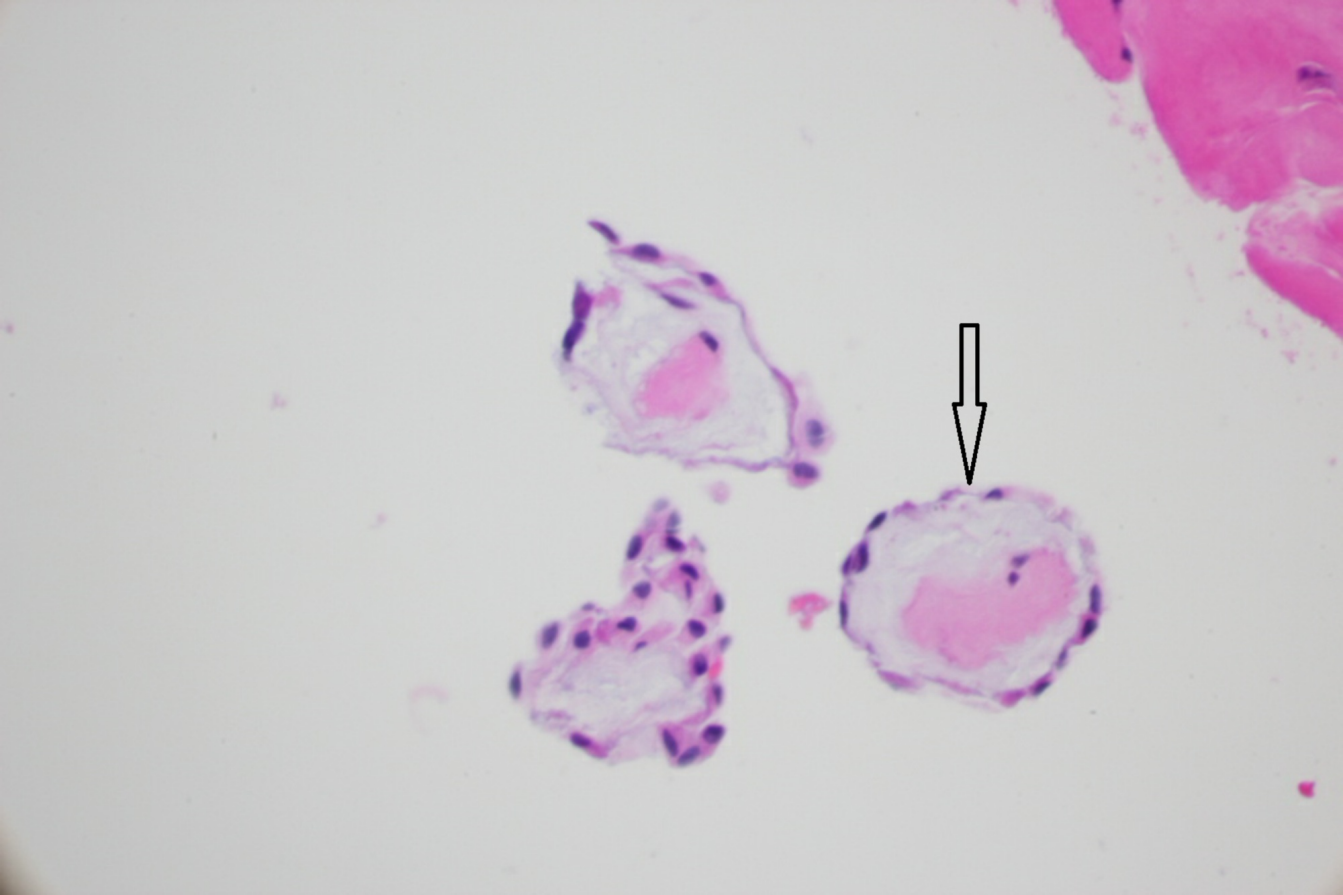
A cross-sectional view of the papillae, with a core of collagen and elastic fibers, lined by the endothelium

There were fibroblasts, collagen, and elastic fibers in the core of papillae (Figure 8). Since then, she has had no recurrence of her symptoms.

## Discussion

Papillary fibroelastoma is a benign cardiac tumor usually found on echocardiography as an incidental finding, or it can present in various ways but rarely presents with infarction of distant organs. Only two cases have been found with splenic infarct, and one case was found to have renal infarct secondary to fibroelastoma [1-2].

Papillary fibroelastoma is the second most common cardiac tumor after cardiac myxoma with a worldwide prevalence ranging from 0.02%-0.45% during autopsies and open heart surgeries, respectively [3-5]. Our search revealed 84 cases on fibroelastoma that have mentioned TIA and stroke being the most common presentation. Emboli were also reported in other locations, including coronary arteries, distal lower limb arteries, and rarely renal and splenic arteries [6]. These tumors cause valvular dysfunction, blockage of the coronary artery, and even sudden death in rare instances. It usually causes embolization, but whether the emboli arise from the overlying thrombus or from the tumor pieces itself is not so clear [7]. The reported embolizations have been associated with TIA or stroke (17%), angina (7%), acute myocardial infarction (3.8%), heart failure (3.3%), sudden cardiac death (3.0%), syncope (1.6%), pulmonary embolism (1%), or blindness (1%) [5]. To the best of our knowledge, there have been two case reports of splenic infarction [1], and one case of the renal infarct [2]. We could not find any case where renal and splenic lesions were found together. Our case is unique in its presentation with an unusual combination of brain, renal, and splenic infarcts due to embolization of cardiac fibroelastoma in the absence of any infective or thrombotic endocarditis (IE). Cardiac fibroelastoma appears pedunculated, flower-like, ranging in size from 2 to 70 mm, with 10 mm being the average size. They usually arise from cardiac valves (85% of cases) with the aortic valve being the most common site (27%) and mitral valve (25%) being the second most common. Other rarely affected valves are tricuspid (17%) and pulmonic valves (15%) [8]. Our patient had a 0.6 x 0.2 x 0.1 cm measuring fibroelastoma attached to the right coronary cusp of the aortic valve.

The most commonly used modality for diagnosis is an echocardiogram [9-12]. Transthoracic echocardiography (TTE) is undoubtedly a useful tool for diagnosis, but most of the lesions need to be picked up by TEE [12]. In our case, the patient had TTE done twice, but the tumor was not seen until the patient underwent TEE. As evident from this case, TTE often fails to detect this tumor. In a case-control study, the sensitivity of TTE for CPF was 61.9%, while that of TEE was 76.6% [8]. Even when abnormal lesions are seen on TTE, it is often difficult to diagnose them as CPF, because of difficulty in identifying the exact site where the tumor is attached to the heart. Other modalities that are inferior to echocardiogram are multispiral CT scan and MRI. Cardiac catheterization is usually not required to make a diagnosis unless other modalities cannot pick the lesion.

The management of CPF depends on the symptoms, location, and size of the mass. In symptomatic cases, the gold-standard treatment is complete resection of the mass. In some cases, valve repair is needed when the tumor is massive and widely attached to the valve [2]. There is no consensus about asymptomatic cases. Regular follow up with clinical examination and echocardiography is recommended in asymptomatic cases. In cases of typical valvular location, immobility, and size 1 cm or less, watchful waiting can be done for asymptomatic people. Tumors more than 1 cm should be excised due to increased risk of embolization and sudden cardiac death [3]. Surgical resection is considered to be curative with no recurrence. For patients at high risk for surgery, long-term anticoagulation is recommended. According to some authors, prophylactic anticoagulation should be started at the time of diagnosis; however, there is no outcome-based data to suggest that this is an ideal approach [5].

## Conclusions

We conclude that CPF can cause embolization to the renal and splenic vasculature, along with cerebral vasculature. Constitutional symptoms may be present along with fibroelastoma. TEE is mandatory if the TTE is negative for suspected cardiac vegetations or mass. Definitive treatment is surgical resection of the lesion. No recurrence has been reported after resection of the fibroelastoma.
